# Short-Term Postpartum Blood Pressure Self-Management and Long-Term Blood Pressure Control: A Randomized Controlled Trial

**DOI:** 10.1161/HYPERTENSIONAHA.120.17101

**Published:** 2021-06-28

**Authors:** Jamie A. Kitt, Rachael L. Fox, Alexandra E. Cairns, Jill Mollison, Holger H. Burchert, Yvonne Kenworthy, Annabelle McCourt, Katie Suriano, Adam J. Lewandowski, Lucy Mackillop, Katherine L. Tucker, Richard J. McManus, Paul Leeson

**Affiliations:** 1 Radcliffe Department of Medicine Division of Cardiovascular Medicine, Cardiovascular Clinical Research Facility (J.K., H.B., Y.K., A.M., K.S., A.J.L., P.L.), University of Oxford, United Kingdom.; 2 Nuffield Department of Primary Care (A.C., J.M., K.T., R.M.), University of Oxford, United Kingdom.; 3 Nuffield Department of Women’s and Reproductive Health (A.C., L.M.), University of Oxford, United Kingdom.; 4 University of Melbourne, Australia (R.F.).; 5 Western Health, Melbourne, Australia (R.F.).

**Keywords:** hypertension, postpartum period, pregnancy, self-management, women

## Abstract

Women with hypertensive pregnancies are 4× more likely to develop chronic hypertension. Previously, we showed a short period of blood pressure (BP) self-management following hypertensive pregnancy resulted in persistently lower BP after 6 months. We now report the impact on long-term BP control. Women who participated in the postpartum randomized controlled trial, SNAP-HT (Self-Management of Postnatal Hypertension; NCT02333240), were invited for 24-hour ambulatory and clinic BP measures. Height and weight were measured by calibrated scales and standardized tape measures, activity by 7-day wrist-worn accelerometer, and dietary factors assessed by questionnaire. Sixty-one of 70 eligible women were followed up 3.6±0.4 years after their original pregnancy. Twenty-four–hour diastolic BP was 7.0 mm Hg lower in those originally randomized to postpartum BP self-management instead of usual care. This difference remained significant after adjustment for either BP at the time of delivery (−7.4 mm Hg [95% CI, −10.7 to −4.2]; *P*<0.001) or pregnancy booking BP (−6.9 mm Hg [95% CI, −10.3 to −3.6]; *P*<0.001). Adjustment for current salt intake, age, body mass index, waist-to-hip ratio, arm circumference, parity, alcohol intake, and physical activity had no effect on this difference. Reductions in diastolic BP at 6 months, following self-management of BP postpartum, are maintained 3.6 years later as measured by lower 24-hour diastolic BP. Interventions to optimize BP control during the puerperium in women with hypertensive pregnancies improve BP in the longer term, in a cohort at increased risk of developing chronic hypertension and major adverse cardiovascular events.

**Registration:**

URL: https://www.clinicaltrials.gov; Unique identifier: NCT02333240.

## Abstract 2

Supplemental Digital Content is available in the text.

Hypertensive disorders of pregnancy, including both gestational hypertension and preeclampsia, affect 10% of pregnancies^1^ and have significant impacts on maternal and fetal health during the peripartum period.^2,3^ Additionally, one-third of women develop chronic hypertension within 10 years^4^ and, over their lifetime, have a 2-fold to 4-fold increased risk of cardiovascular disease,^5^ stroke, and renal dysfunction.^6^ As a result, the US and European guidelines^7,8^ advise that women should be informed of this higher incidence of disease after pregnancy and advise annual blood pressure (BP) monitoring, but, beyond routine risk factor management, no specific additional interventions are currently available to reduce long-term risk.^9^

Prior research shows that persistently high BP 6 weeks after a hypertensive pregnancy is associated with a greater increase in BP over the next 5 to 10 years.^10^ This is independent of BP levels during early pregnancy, or at time of delivery, and is evident both in those with more severe hypertensive disorders of pregnancy, such as early-onset preeclampsia and later onset, milder disease. A plausible explanation might be that BP at 6 weeks postpartum is merely a marker of BP trajectory over the life course for that individual.^11^ However, without intervention, BP remains unstable for up to 50% of women for several months after a hypertensive pregnancy.^12,13^ An alternative explanation is that uncontrolled BP in the puerperium directly affects the cardiac and vascular remodeling, known to occur in the weeks after a hypertensive pregnancy in both mother and child.^14–18^ In turn, this may lead to a long-term persistence of elevated BP, accelerating a lifelong trajectory of increased vascular risk.

To test this hypothesis required a randomized controlled trial of BP optimization after a hypertensive pregnancy. Trials of self-management, outside of pregnancy, have repeatedly demonstrated improved BP control.^19–24^ In the SNAP-HT trial (Self-Management of Postnatal Hypertension),^25^ our group confirmed that postpartum self-management was feasible and that it improved BP control, with a more rapid return to normal BP ranges within 6 weeks of delivery, when compared with usual care. In addition, 6 months later, those randomized to postpartum self-management had a persistent 4.5-mm Hg reduction in diastolic BP.^25^ We have now performed a long-term follow-up of this cohort (SNAP-HT Extension Study), to formally test the hypothesis that interventions to improve BP over the first few months following a hypertensive pregnancy result in long-term reprogramming of BP in the mother.

## Methods

### Recruitment and Enrollment

This was a prospective long-term follow-up of a prior unmasked, randomized trial (SNAP-HT; Research Ethics Committee reference 14/SC/1316; NCT02333240) using a cohort of women who had preeclampsia or gestational hypertension and who required BP-lowering medication in the postpartum period. In the original SNAP-HT trial, women were randomized to usual care (control arm) or BP self-management (the intervention). After discharge from hospital, women in the usual care cohort visited their community midwife for BP monitoring and their general practitioner for titration of antihypertensive medication. Women allocated to the self-management arm of the trial self-monitored their BP once daily, at approximately the same time each day, using a validated automatic BP monitor (WATCHBPHOME monitors by Microlife). BP readings were entered by participants into their mobile phone and automatically transmitted to the study server. In response, the telemonitoring service provided automated replies that informed women how to downtitrate their medications, according to a prespecified individualized medication reduction schedule. If readings were high, according to an algorithm based on National Institute for Health and Care Excellence guidance at that time (CG107),^13^ participants were asked to contact their National Health Service care team immediately. Self-monitoring was continued while taking antihypertensive treatment and for 5 days after treatment was ceased. Once treatment was ceased, if the participant’s BP remained within normal limits for 5 consecutive days, they were instructed to change to weekly self-monitoring until the trial was completed to ensure medication did not need to be restarted if readings moved out of the target range again.^25^

Participants in the original SNAP-HT trial were recruited from 5 National Health Service hospital sites in England and at the time of the study, were asked whether they were willing to give consent to be contacted about future research projects. All participants recruited to the Extension Study were asked to give permission to access the data from the original trial. A participant was ineligible if they were pregnant at the time of reassessment or they had a significant new comorbidity that made enrollment unsafe.

Members of the original SNAP-HT research team (A.C. and L.M.) made contact with eligible participants, and once verbal consent was obtained, the SNAP-HT Extension team performed telephone screening for eligibility according to the inclusion and exclusion criteria. Women then participated in either a home visit or a single study visit to the Oxford Cardiovascular Clinical Research Facility in the John Radcliffe Hospital, based on participant preference.

This study was prospectively registered and approved by a National Research Ethics Service Committee (19/SW/0017). The data that support the findings of this study are available from the corresponding author on reasonable request.

### Study Measurements

BP was measured 6× using a standardized method, after 5 minutes of rest, at 1-minute intervals using the same Microlife WATCHBP monitors used in the original SNAP-HT trial.^25^ These were serviced and calibrated before use and are validated for use in pregnancy and preeclampsia.^26^ All participants had their arm circumference measured, and the appropriate cuff size was used. All researchers were trained to use the monitors. The systolic BP, diastolic BP, and mean arterial pressure were recorded.

In addition, current medications, side effects, serious adverse events, and quality-of-life scores EuroQol- 5 Dimension-5 Level were recorded. A detailed questionnaire was completed to identify possible lifestyle and dietary factors or factors in their social background that could confound any BP differences detected in the study. This included a validated British Heart Foundation diet and lifestyle questionnaire.^27^ Height and weight were recorded using standardized, calibrated weighing scales and tape measures.

Participants were then fitted with calibrated and serviced SPACELABS 90217 or 90217A 24-hour ambulatory BP monitors at the end of their study visit using a cuff that had been measured to appropriately fit their mid-left arm circumference. The monitors were programmed to take an automated measurement at 30-minute intervals between 8 AM and 10 PM and 60-minute intervals from 10 PM to 8 AM. To obtain a validated objective measure of physical activity, participants were fitted with Axivity AX3 wrist-worn accelerometer for 7 days to ensure high compliance and measurement reliability.

Twenty-four–hour BP data were analyzed as per National Institute for Health and Care Excellence NG 136 criteria.^28^ Any manual measurements made at the time of monitor fitting were excluded, as were any measurements obtained at a time of documented prespecified form of exercise on the patient’s diary. Twenty-four–hour BP data were only included if an average value of at least 14 measurements taken during the person’s usual waking hours was obtained. If the recording did not fulfill these criteria,^29^ then the 24-hour BP recording was repeated with consent at the next available opportunity.

### Statistical Analyses

The prespecified primary outcome of the SNAP-HT Extension Study was 24-hour average diastolic BP as assessed by 24-hour ambulatory blood pressure monitoring. Secondary outcomes included 24-hour average systolic BP, mean diurnal diastolic BP, mean diurnal systolic BP, mean nocturnal diastolic BP, and mean nocturnal systolic BP. As per the original SNAP-HT trial, mean clinic systolic BP and diastolic BP obtained during the study visit (mean of the second and third readings and of the second to sixth readings) were also explored as secondary outcomes, as well as mean EuroQol Visual Analogue Scale scores between groups.

Dietary and lifestyle factors known to influence hypertensive risk were also assessed, including salt intake, alcohol consumption, body mass index (BMI), smoking history, arm circumference, waist-to-hip ratio measurements, and objective measurements of activity level from the 7-day wrist-worn accelerometer.

Analysis was conducted according to a statistical analysis plan prepared before analysis. Data obtained in the SNAP-HT Extension Study were linked back to the original SNAP-HT data, with participant consent. Participants in the original trial had been randomized by computer-generated and secure web-based randomization software to ensure allocation concealment. In this study, the investigators were blinded to this allocation until data collection and analysis was complete. Analyses were performed with IBM SPSS Statistics, version 26.

An intention-to-treat approach was taken without imputation for missing data. A small number of participants (n=3) allocated to the usual care arm had consented to participate in SNAP-HT during pregnancy but then were not eligible to be randomized following birth as their antihypertensive medication was stopped. A sensitivity analysis excluded these 3 participants. Continuous variables were reported as mean±SDs or medians with interquartile ranges if skewed. Categorical variables were reported as counts with percentages. Differences between groups were reported with 95% CIs and significance tests with *P*.

Ambulatory (24 hours) and clinic BP readings (the mean of the second and third and of the second to sixth readings) were used for the main analysis. A small number of participants (n=2) were identified as having started on antihypertensive medication and, in view of the effect of medication on average BP levels, were excluded from analysis. Linear regression modeling fitted with treatment group as the independent variable was used to evaluate differences in BP between the self-management and usual care groups. Primary and secondary BP outcomes were adjusted for the BP readings from the original SNAP-HT baseline visit (taken between the first and sixth day postpartum), in which a difference in BP between the self-management and usual care groups was seen in the original SNAP-HT paper.^25^ This adjustment was performed using 3 models; the first adjusted for the mean of the second and third BP readings at the SNAP-HT baseline visit, as this adjustment was used in the original SNAP-HT analysis. The first sensitivity analysis adjusted for the mean of the second to sixth BP readings at the SNAP-HT baseline visit, as this is felt to better represent a mean ambulatory BP. A second sensitivity analysis adjusted for antenatal BP readings, to assess the impact of prepregnancy BP, was performed based on the antenatal booking visit.^30^ Due to a small amount of missing baseline (n=3) and antenatal (n=3) BP readings, a sensitivity analysis was also performed replacing missing covariate data with mean imputation.

Exploratory analyses were conducted to assess the effect of potential confounding factors (age, parity, BMI, activity, smoking, alcohol intake, salt intake, arm circumference, and waist-to-hip ratio) on 24-hour diastolic BP. These variables were fitted as independent variables in univariable linear regression models and if found to significantly (*P*<0.05) influence 24-hour diastolic BP, were included in a multiple regression analysis with treatment group and baseline BP.

## Results

**Figure 1.**
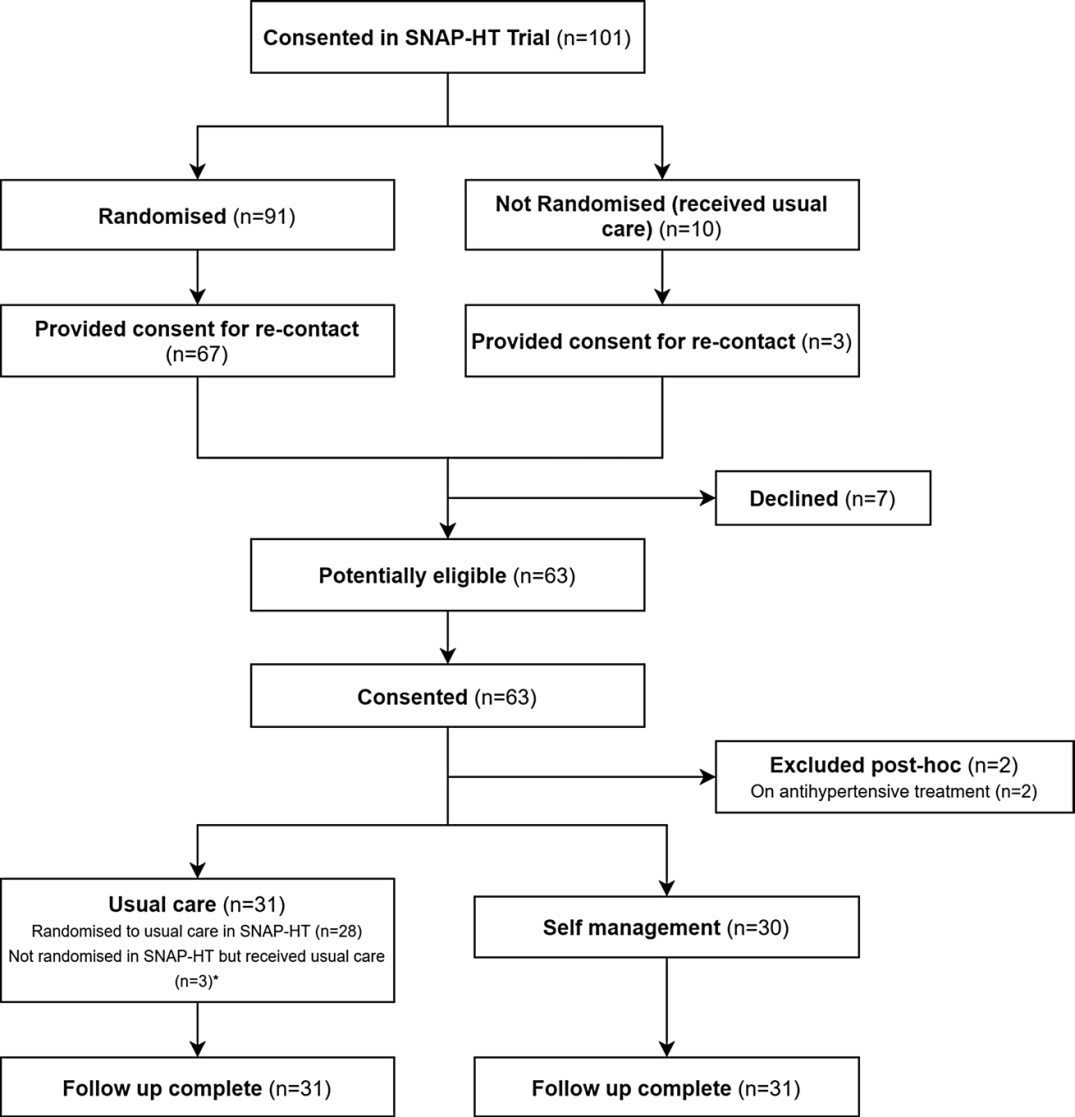
**Consolidated Standards of Reporting Trials flowchart.** *The usual care group included 3 women originally consented in SNAP-HT (Self-Management of Postnatal Hypertension) antenatally but who withdrew before randomization and did not receive the intervention. They were included in the usual care group, as this was felt to be consistent with their postpartum follow-up.

Of the 101 women recruited to the original SNAP-HT trial, 70 provided consent for recontact regarding future studies. All 70 were approached between April 1, 2019, and December 1, 2019, and 63 agreed to participate in this Extension Study. Two women in the usual care group were excluded from the data analysis post hoc as they reported antihypertensive medication on their health questionnaire. The remaining 61 women included in the analysis were not on antihypertensive treatment. Of these 61, 31 received usual care and 30 were randomized to self-management. A Consolidated Standards of Reporting Trials diagram is provided in Figure 1. Follow-up occurred at 3.6±0.4 years postpartum.

### Study Population Characteristics

Table 1 demonstrates that pregnancy characteristics did not differ significantly between participants in the SNAP-HT Extension Study according to whether they originally received self-management or usual care. Table 1 also demonstrates that mean age, BMI, parity, activity levels, mean arm circumference, and EuroQol Visual Analogue Scale scores were similar between self-management (intervention) and usual care (control) groups at the time of their participation in the SNAP-HT Extension Study. Twenty-two women had one subsequent pregnancy in the intervening years between the original trial and follow-up (usual care, n=12; self-management, n=10), and 2 women had 2 pregnancies (one in each group) during this interval (Table 1). Rates of pregnancy hypertension were similar between groups. There were 4 current smokers, all in the usual care group. Baseline characteristics of participants included in the SNAP-HT Extension Study analysis were similar to the original SNAP-HT participants, as well as those participants who were not re-recruited/excluded (Table S1 in the Data Supplement).

**Table 1.**
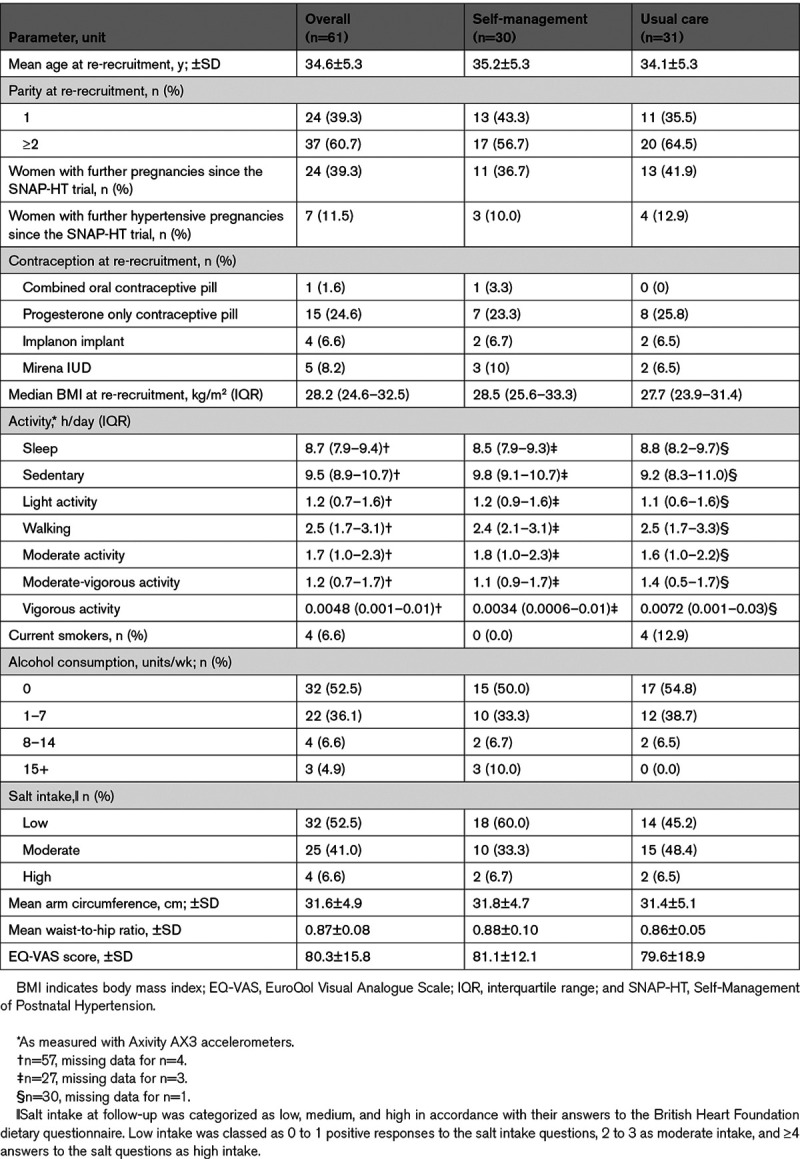
Characteristics of Each Study Group in SNAP-HT Extension at Time of Re-Recruitment (Mean, 43.4 mo Postpartum)

### Longer Term BP Postpartum

**Figure 2.**
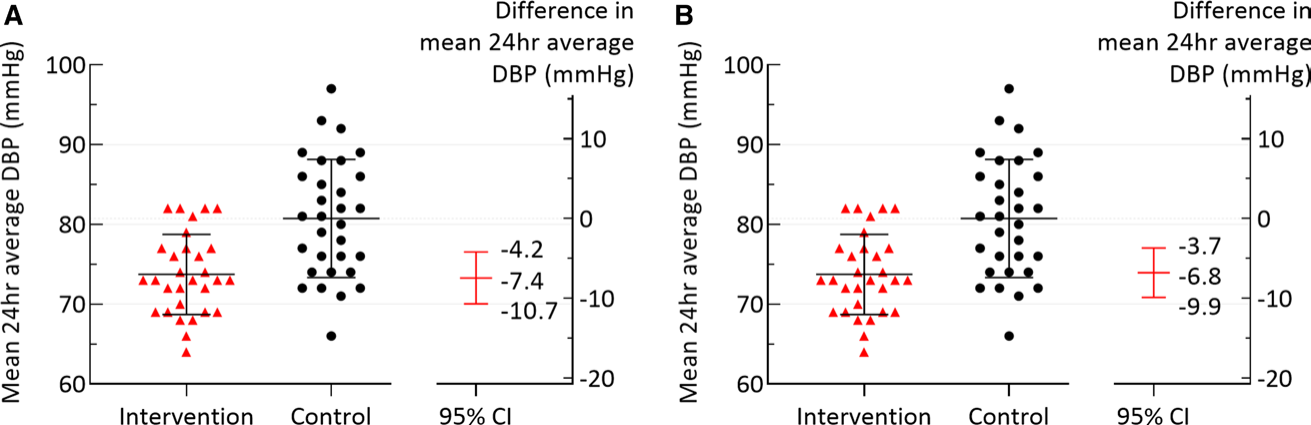
**Primary outcome 24 hour ambulatory blood pressure.** **A**, Primary outcome adjusted for baseline blood pressure in SNAP-HT (Self-Management of Postnatal Hypertension; mean of second and third readings). **B**, Primary outcome adjusted for baseline blood pressure (mean of second and third) and salt intake at 4 y postpartum. DBP indicates diastolic blood pressure.

Women who self-managed their BP postpartum in the SNAP-HT randomized controlled trial had a lower 24-hour diastolic BP of 73.7±5.0 mm Hg compared with 80.7±7.4 mm Hg in the usual care group (adjusted mean difference, −7.4 mm Hg [95% CI, −10.7 to −4.2]; *P*<0.001; Table 2; Figure 2A). Results were similar when nonrandomized participants were excluded from the analysis with the adjusted mean difference then being −7.4 mm Hg ([95% CI, −10.5 to −4.4] *P*<0.001; Table S4). These participants had baseline data but were not randomized in the original trial due to their medication being stopped before hospital discharge in the original SNAP-HT trial. Results also remain similar when missing covariate data were replaced with mean imputation (adjusted mean difference, −7.3 mm Hg [95% CI, −10.4 to −4.1]; *P*<0.001). Additionally, in sensitivity analyses, the difference in 24-hour diastolic BP between the cohorts was maintained when adjusting for baseline diastolic BP using the mean of second to sixth readings (adjusted mean difference, −7.3 mm Hg [95% CI, −10.5 to −4.0]; *P*<0.001) or the antenatal diastolic BP (adjusted mean difference, −6.9 mm Hg [95% CI, −10.3 to −3.6]; *P*<0.001; Table S3).

**Table 2.**
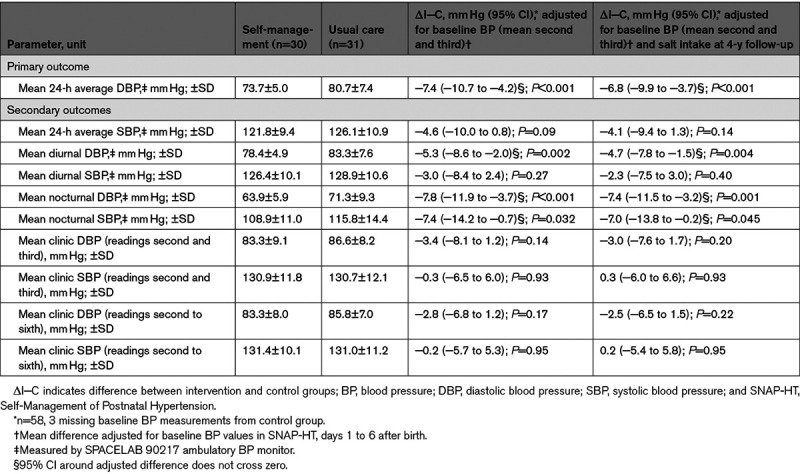
Summary Results for the SNAP-HT Extension Study

In sensitivity analyses, to explore other factors known to influence BP including age, parity, BMI, activity levels, smoking rates, alcohol intake, waist-to-hip ratio, and arm circumference, only salt intake showed a significant association with 24-hour diastolic BP (Table S2). However, the difference in 24-hour diastolic BP in those randomized to self-management remained after adjustment for salt intake at follow-up (adjusted mean difference, −6.8 mm Hg [95% CI, −9.9 to −3.7]; *P*<0.001; Table 2; Figure 2B).

On analysis of the secondary outcomes (Table 2), diurnal diastolic BP was 5.3 mm Hg lower in the self-management group ([95% CI, −8.6 to −2.0] *P*=0.002), and both nocturnal diastolic and systolic BP were significantly lower in the self-management group (adjusted mean difference: nocturnal diastolic BP, −7.8 mm Hg [95% CI, −11.9 to −3.7]; *P*<0.001 and nocturnal systolic BP, −7.4 [95% CI, −14.2 to −0.7]; *P*=0.032). Twenty-four–hour and diurnal systolic BP did not significantly differ. Clinic diastolic BP and systolic BP readings, both the mean of second and third and of second to sixth, did not significantly differ between groups (Table 2).

## Discussion

This study demonstrates that 3 to 4 years after a hypertensive pregnancy, women who received a short period of postpartum BP self-management had persistently lower diastolic BP, consistent with a long-term impact of the original intervention, resetting BP levels.

The original SNAP-HT trial was designed to assess feasibility and safety of a self-management digital intervention in the postnatal period, but exploring the impact of the intervention on BP was an important secondary outcome.^25^ The finding in SNAP-HT of a persistently lower diastolic BP at 6 months, when almost all women had stopped treatment, prompted the development of the current study. The primary aim of the SNAP-HT Extension Study was to assess whether this reduction in diastolic BP persisted several years after the original intervention had finished. In the intervening period, traditional hypertensive risk factors might have diluted any ongoing BP benefit,^31^ with additional regression to the mean possible. To allow evaluation of this potential effect, data on salt and alcohol intake, as well as objective measures of exercise, BMI, and smoking, were collected. Ultimately, in analysis, only salt intake was found to have a significant impact on BP differences between groups, albeit with the significant diastolic difference between groups remaining following adjustment. Differences were also not explained by variation in contraception use between groups.

In the original trial, the number of antihypertensives prescribed, as assessed by the median (interquartile range) World Health Organization–defined daily dose,^32^ was similar in the self-management and usual care cohort at 4 and 6 weeks,^25^ (World Health Organization–defined daily dose at 4 weeks: 0.4 [0–1] in usual care and 0.3 [0–0.7] in self-management; 6 weeks: 0.4 [0–0.8] and 0.2 [0–0.5]). Antihypertensive treatment in the original trial was set by the treating clinical team and agents used included labetalol, captopril, enalapril, nifedipine, amlodipine, and methyldopa. Medications were reduced/ceased in the order: α-blockers, β-blockers, calcium channel blockers, and then angiotensin converting enzyme inhibitors, as per the National Institute for Health and Care Excellence essential hypertension guidelines at that time,^13^ but in reverse. As reported in the original trial publication,^25^ there were no significant differences in the class of medications being used in each treatment arm. Therefore, it is unlikely that effects of self-management on BP in the intervention group were mediated through greater prescription of antihypertensives but, consistent with previous reports on the impact of self-management on BP control,^33^ are more likely to relate to greater medication adherence. Furthermore, during the SNAP-HT trial, more women were readmitted to hospital for high BP (systolic >150 or diastolic >100 mm Hg) and had their medications uptitrated promptly before discharge (4 in self-management arm and 1 in usual care). This difference, albeit in small numbers, suggests opportunities may have been missed to uptitrate medication in the usual care arm. In summary, although the class and dose of antihypertensive used did not differ between trial arms, those in the intervention arm likely had greater exposure to the antihypertensive medication, which may have aided remodeling postpartum.

A 7- to 8-mm Hg difference in 24-hour ambulatory diastolic BP would be expected to reduce cardiovascular risk by >30% and, thereby, have a meaningful impact on the increased incidence of cardiovascular and cerebrovascular disease seen following a hypertensive pregnancy.^34^ Indeed, 2 women who originally received usual care following their hypertensive pregnancy had already developed chronic hypertension requiring ongoing treatment. A specific effect on diastolic pressure is consistent with recent longitudinal cohort data, starting from prepregnancy.^30^ In the Trøndelag Health study cohort, it was observed that diastolic BP did not return to prepregnancy levels postpartum in those with hypertensive pregnancies, in contrast to the pattern seen in those with normotensive pregnancies. Our long-term follow-up of a randomized trial suggests this natural history of disease progression after a hypertensive pregnancy may not be fixed but, rather, a potentially modifiable phenomenon.^30^ In the intervening years between the original trial and follow-up, 40% of women had a further pregnancy. Although rates of hypertensive pregnancy were similar between groups, further work, in a larger sample, is required to understand whether a persistent 7- to 8-mm Hg reduction in BP could also result in a meaningful reduction in risk of pregnancy hypertension within the population in subsequent pregnancies.

The study was designed to utilize ambulatory BP measures to improve precision of the BP estimate in a small sample size and maximize power to identify differences between groups.^35–37^ Reassuringly, differences in diastolic BP were evident on both diurnal and nocturnal ambulatory measures, and the difference in ambulatory measures identified was in fact greater than the 4.5-mm Hg difference in clinic diastolic BP recorded at 6 months. However, the differences in clinic measures seen in this Extension Study were not statistically significant. This may reflect a lack of power due to the inherent variability in clinic BPs within a small sample, evidenced by the larger SDs (12.1 mm Hg in the usual care arm and 11.8 mm Hg in the self-management arm for clinic versus 7.4 and 5 mm Hg, respectively, utilizing ambulatory blood pressure monitoring). An alternative explanation is that it was a general alerting/white coat response, which masked differences in the clinic BP readings.

Why a permanent change in BP can be induced by a short-term postpartum intervention remains under investigation in ongoing studies by our group, and others, but it may relate to cardiovascular adaptations associated with the hypertensive pregnancy.^38,39^ Normal pregnancy is associated with substantial cardiac and vascular remodeling.^39^ The addition of acute inflammatory, antiangiogenic, and oxidative stresses of a hypertensive pregnancy results in further significant adverse changes.^40,41^ Persistence of left ventricular hypertrophy at 1 year postpartum in women with preterm preeclampsia was strongly associated with risk of developing essential hypertension within the subsequent 2 years.^38,42^ We have previously described left ventricular hypertrophy, diastolic dysfunction, capillary rarefaction, and reduced aortic compliance^17^ at 5 to 10 years following a hypertensive pregnancy, compared with a normotensive pregnancy. We hypothesize that the long-term improvements in BP we have observed may reflect a regression of this adverse remodeling and are currently testing this hypothesis in the Physician Optimised Post-Partum Hypertension Treatment Trial (https://clinicaltrials.gov/ct2/show/NCT04273854).

The hypothesis that improved BP control during the puerperal could have long-term benefits has recently been supported by findings from a randomized trial of postpartum enalapril, in addition to usual BP care. This resulted in lower BP at 6 months postpartum, even after the enalapril had been withdrawn.^43^ Furthermore, women receiving enalapril had improved diastolic function and left ventricular remodeling compared with placebo.

The SNAP-HT extension participants were originally recruited into a feasibility study and, although we did manage to recruit 90% of the potentially eligible participants, the conclusions are limited by the small sample size of the original trial population. In addition, the participants were largely white and tended to be from higher socioeconomic classes, which limits our ability to generalize our findings to the broader population. Hypertensive risk factors were only assessed at one time point, 3 to 4 years postpartum, and may not reflect the lifestyle characteristics of the participants throughout this entire period. Furthermore, while activity levels, BMI, arm circumference, and waist-to-hip ratio were measured objectively, lifestyle factors relied on participant self-reporting, which may influence the accuracy of these results.^44^ Fifty-nine of 61 visits were conducted at the participants’ homes with only 2 at the John Radcliffe Hospital. Therefore, any possible effect of the clinic setting on BP measures is likely to be too small to influence the results. Moreover, adjustment for baseline BPs needs to be viewed cautiously as this relied on averaged clinic readings at baseline in contrast to the 24-hour ambulatory BP readings in the current study.

### Perspectives

Up to 10% of pregnancies are affected by new-onset hypertension, and women who have had a hypertensive pregnancy are at substantially increased risk of developing hypertension and cardiovascular disease in later life. This study shows short-term self-management of BP, designed to optimize BP control immediately after hypertensive pregnancies, appears to reset BP levels over longer time periods. High BP is the leading risk factor for loss of disability-adjusted life-years in high- and low- to middle-income countries and short, targeted postpartum interventions could offer a clinically translatable approach to improve BP outcomes for women within this population. Self-management of BP has been shown to be cost-effective in other clinical scenarios^45^ and due to its patient acceptability and ease of use in remote health care, has been proposed in recent national guidance^46^ for provision of postpartum care during the coronavirus disease 2019 (COVID-19) pandemic. Our research provides a supportive evidence base that this step change in postpartum practice might also have longer term benefits for reducing cardiovascular disease burden within the population.^47^

### Conclusions

This study has shown in a randomized trial that it is possible to lower BP for at least 3 to 4 years postpartum by ensuring tighter BP control during the postpartum period while women require treatment. The findings support the hypothesis developed from prior observational work by our group that a failure to control BP postpartum leads to an accelerated trajectory toward hypertension over subsequent years, possibly due to failure to reverse the adaptive cardiovascular remodeling induced by a hypertensive pregnancy.^10^ Critically, this study highlights that there may be a window postpartum where a relatively affordable and clinically translatable process of home BP self-management can impact long-term BP trajectory for women after a hypertensive pregnancy and reduce the later burden of cardiovascular and cerebrovascular disease.

**Figure 3.**
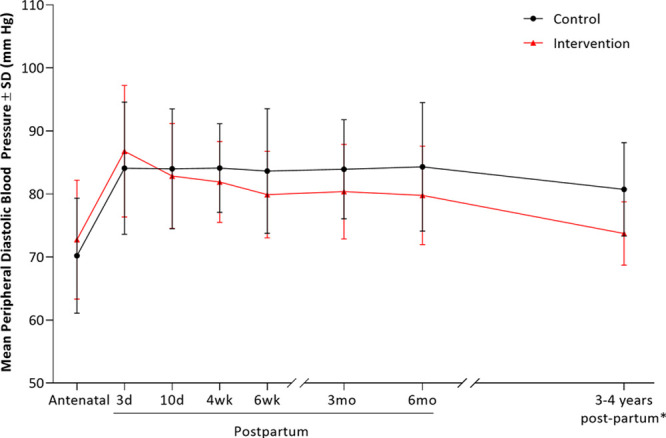
**Longitudinal diastolic blood pressure data for SNAP-HT (Self-Management of Postnatal Hypertension) and SNAP-HT^25^ Extension from antenatal booking blood pressure to 3 to 4 y postpartum demonstrating the long-term benefit of self-management on blood pressure control following a hypertensive pregnancy.** *Blood pressure at 3 to 4 y postpartum is average 24-h diastolic blood pressure as measured by ambulatory blood pressure monitor.

## Acknowledgments

The views expressed are those of the authors and not necessarily those of the National Health Service (NHS), the National Institute for Health Research, or the Department of Health. This research would not have been possible without the help of the participants. We would also like to extend particular thanks to the Ethics Committee and to Lucy Chappell and Baskaran Thilaganathan for their support throughout. We also give thanks to the original SNAP-HT (Self-Management of Postnatal Hypertension) authors not included in the Extension Study: Mauro Santos, Carmelo Velardo, Dario Salvi, Sam Mort, and Lionel Tarassenko. We also give thanks to the original SNAP-HT investigators, who in addition to those listed as authors were as follows: Carole Crawford (Research Midwife), University of Oxford; Claire Edwards (Research Midwife), University of Oxford; Natasha Baker (Research Midwife) and Mark Selinger (Principal Investigator), Royal Berkshire NHS Foundation Trust; Sue Lloyd (Principal Investigator), Northampton General Hospital NHS Trust; Julie Tebbutt (Research Midwife) and Felicity Ashworth (Principal Investigator), Buckinghamshire Healthcare NHS Trust; and Rebecca Pullon (Doctoral Student), University of Oxford. R. McManus gained funding for the study. J. Kitt led the study, drafted the report, and undertook the analyses, with statistical work led by R. Fox and guidance from J. Mollison. All authors participated in design, execution, and oversight of the study. All authors had access to the data, commented on subsequent drafts, and approved the final submitted version. R. McManus and P. Leeson will act as guarantors and with J. Kitt made the final decision to submit for publication. We also thank Aiden Doherty and Shing Chan from the Nuffield Department of Population Health for their vital assistance in the analysis of the accelerometer data.

## Sources of Funding

R. McManus and P. Leeson acknowledge support from the National Institute for Health Research (NIHR) Oxford Collaboration for Leadership in Health Research and Care. J. Kitt is funded by a British Heart Foundation Clinical Research Training Fellowship (British Heart Foundation grant number FS/19/7/34148). P. Leeson acknowledges support from the Oxford British Heart Foundation Centre for Research Excellence and NIHR Oxford Biomedical Research Centre. K. Tucker and R. McManus are funded by an NIHR programme grant for applied research (RP-PG-0614-20005) and have received funding from the NIHR Applied Research Collaboration Oxford and Thames Valley at Oxford Health National Health Service Foundation Trust. R. McManus is an NIHR Senior Investigator.

## Disclosures

L. Mackillop is supported by the National Institute for Health Research Oxford Biomedical Research Centre and is a part-time employee of Sensyne Health plc and holds shares in this company. R. McManus has received blood pressure monitors for research from Omron and is working with them to develop a telemonitoring system. Any fees/consultancy from this work is paid to his institution. The other authors report no conflicts.

## Supplemental Materials

Data Supplement Tables S1–S4

## Supplementary Material



## Novelty and Significance

### What Is New?

- Self-management of blood pressure (via blood pressure self-monitoring and subsequent medication titration) after a hypertensive pregnancy results in a persistent reduction in diastolic blood pressure by 7 mm Hg at 3 to 4 years postpartum.

### What Is Relevant?

- Women with hypertensives pregnancies are at an increased risk of cardiovascular disease later in life. A 7- to 8-mm Hg reduction in blood pressure would be expected to reduce cardiovascular risk by >30%.

### Summary

In women with hypertension in pregnancies, short-term optimization of blood pressure postpartum appears to reset blood pressure levels in the longer term.

## References

[1] Ananth CV Keyes KM Wapner RJ. Pre-eclampsia rates in the united states, 1980-2010: age-period-cohort analysis. BMJ. 2013;347:f6564 24201165 10.1136/bmj.f6564 PMC3898425

[2] Ditisheim A Wuerzner G Ponte B Vial Y Irion O Burnier M Boulvain M Pechère-Bertschi A. Prevalence of hypertensive phenotypes after preeclampsia: a prospective cohort study. Hypertension. 2018;71:103–109. doi: 10.1161/HYPERTENSIONAHA.117.09799 29133363 10.1161/HYPERTENSIONAHA.117.09799

[3] Aye CYL Lewandowski AJ Lamata P Upton R Davis E Ohuma EO Kenworthy Y Boardman H Frost AL Adwani S. Prenatal and postnatal cardiac development in offspring of hypertensive pregnancies. J Am Heart Assoc. 2020;9:e014586. doi: 10.1161/JAHA.119.014586 32349586 10.1161/JAHA.119.014586 PMC7428573

[4] Behrens I Basit S Melbye M Lykke JA Wohlfahrt J Bundgaard H Thilaganathan B Boyd HA. Risk of post-pregnancy hypertension in women with a history of hypertensive disorders of pregnancy: nationwide cohort study. BMJ. 2017;358:j3078. doi: 10.1136/bmj.j3078 28701333 10.1136/bmj.j3078 PMC5506851

[5] McDonald SD Malinowski A Zhou Q Yusuf S Devereaux PJ. Cardiovascular sequelae of preeclampsia/eclampsia: a systematic review and meta-analyses. Am Heart J. 2008;156:918–930. doi: 10.1016/j.ahj.2008.06.042 19061708 10.1016/j.ahj.2008.06.042

[6] Ray JG Vermeulen MJ Schull MJ Redelmeier DA. Cardiovascular health after maternal placental syndromes (CHAMPS): population-based retrospective cohort study. Lancet. 2005;366:1797–1803. doi: 10.1016/S0140-6736(05)67726-4 16298217 10.1016/S0140-6736(05)67726-4

[7] Meschia JF Bushnell C Boden-Albala B Braun LT Bravata DM Chaturvedi S Creager MA Eckel RH Elkind MS Fornage M; American Heart Association Stroke Council; Council on Cardiovascular and Stroke Nursing; Council on Clinical Cardiology; Council on Functional Genomics and Translational Biology; Council on Hypertension. Guidelines for the primary prevention of stroke: a statement for healthcare professionals from the American Heart Association/American Stroke Association. Stroke. 2014;45:3754–3832. doi: 10.1161/STR.0000000000000046 25355838 10.1161/STR.0000000000000046 PMC5020564

[8] Regitz-Zagrosek V Blomstrom Lundqvist C Borghi C Cifkova R Ferreira R Foidart JM Gibbs JS Gohlke-Baerwolf C Gorenek B Iung B. European Society of Gynecology; Association for European Paediatric Cardiology; German Society for Gender Medicine. ESC Committee for Practice Guidelines. ESC guidelines on the management of cardiovascular diseases during pregnancy: the task force on the management of cardiovascular diseases during pregnancy of the European Society of Cardiology (ESC). Eur Heart J. 2011;32:3147–3197. doi: 10.1093/eurheartj/ehr218 21873418 10.1093/eurheartj/ehr218

[9] Cairns AE Pealing L Duffy JMN Roberts N Tucker KL Leeson P MacKillop LH McManus RJ. Postpartum management of hypertensive disorders of pregnancy: a systematic review. BMJ Open. 2017;7:e018696. doi: 10.1136/bmjopen-2017-018696 10.1136/bmjopen-2017-018696 PMC5719299 29187414

[10] Lazdam M de la Horra A Diesch J Kenworthy Y Davis E Lewandowski AJ Szmigielski C Shore A Mackillop L Kharbanda R. Unique blood pressure characteristics in mother and offspring after early onset preeclampsia. Hypertension. 2012;60:1338–1345. doi: 10.1161/HYPERTENSIONAHA.112.198366 23045462 10.1161/HYPERTENSIONAHA.112.198366

[11] Lazdam M Davis EF Lewandowski AJ Worton SA Kenworthy Y Kelly B Leeson P. Prevention of vascular dysfunction after preeclampsia: a potential long-term outcome measure and an emerging goal for treatment. J Pregnancy. 2012;2012:704146. doi: 10.1155/2012/704146 22175025 10.1155/2012/704146 PMC3235810

[12] Walters BN Walters T. Hypertension in the puerperium. Lancet. 1987;2:330. doi: 10.1016/s0140-6736(87)90912-3 10.1016/s0140-6736(87)90912-3 2886783

[13] National Institute for Clinical Excellence (NICE). Clinical Guideline 107 (CG107). 2011. https://www.nice.org.uk/guidance/cg107

[14] Siepmann T Boardman H Bilderbeck A Griffanti L Kenworthy Y Zwager C McKean D Francis J Neubauer S Yu GZ. Long-term cerebral white and gray matter changes after preeclampsia. Neurology. 2017;88:1256–1264. doi: 10.1212/WNL.0000000000003765 28235810 10.1212/WNL.0000000000003765 PMC5373775

[15] Lewandowski AJ Augustine D Lamata P Davis EF Lazdam M Francis J McCormick K Wilkinson AR Singhal A Lucas A. Preterm heart in adult life: cardiovascular magnetic resonance reveals distinct differences in left ventricular mass, geometry, and function. Circulation. 2013;127:197–206. doi: 10.1161/CIRCULATIONAHA.112.126920 23224059 10.1161/CIRCULATIONAHA.112.126920

[16] Davis EF Lazdam M Lewandowski AJ Worton SA Kelly B Kenworthy Y Adwani S Wilkinson AR McCormick K Sargent I. Cardiovascular risk factors in children and young adults born to preeclamptic pregnancies: a systematic review. Pediatrics. 2012;129:e1552–e1561. doi: 10.1542/peds.2011-3093 22614768 10.1542/peds.2011-3093

[17] Boardman H Lamata P Lazdam M Verburg A Siepmann T Upton R Bilderbeck A Dore R Smedley C Kenworthy Y. Variations in cardiovascular structure, function, and geometry in midlife associated with a history of hypertensive pregnancy. Hypertension. 2020;75:1542–1550. doi: 10.1161/HYPERTENSIONAHA.119.14530 32306767 10.1161/HYPERTENSIONAHA.119.14530 PMC7682801

[18] Yu GZ Aye CY Lewandowski AJ Davis EF Khoo CP Newton L Yang CT Al Haj Zen A Simpson LJ O’Brien K. Association of maternal antiangiogenic profile at birth with early postnatal loss of microvascular density in offspring of hypertensive pregnancies. Hypertension. 2016;68:749–759. doi: 10.1161/HYPERTENSIONAHA.116.07586 27456522 10.1161/HYPERTENSIONAHA.116.07586 PMC4978605

[19] McLean G Band R Saunderson K Hanlon P Murray E Little P McManus RJ Yardley L Mair FS; DIPSS Co-Investigators. Digital interventions to promote self-management in adults with hypertension systematic review and meta-analysis. J Hypertens. 2016;34:600–612. doi: 10.1097/HJH.0000000000000859 26845284 10.1097/HJH.0000000000000859 PMC4947544

[20] McManus RJ Mant J Bray EP Holder R Jones MI Greenfield S Kaambwa B Banting M Bryan S Little P. Telemonitoring and self-management in the control of hypertension (TASMINH2): a randomised controlled trial. Lancet. 2010;376:163–172. doi: 10.1016/S0140-6736(10)60964-6 20619448 10.1016/S0140-6736(10)60964-6

[21] McManus RJ Mant J Franssen M Nickless A Schwartz C Hodgkinson J Bradburn P Farmer A Grant S Greenfield SM; TASMINH4 Investigators. Efficacy of self-monitored blood pressure, with or without telemonitoring, for titration of antihypertensive medication (TASMINH4): an unmasked randomised controlled trial. Lancet. 2018;391:949–959. doi: 10.1016/S0140-6736(18)30309-X 29499873 10.1016/S0140-6736(18)30309-X PMC5854463

[22] McManus RJ Mant J Haque MS Bray EP Bryan S Greenfield SM Jones MI Jowett S Little P Penaloza C. Effect of self-monitoring and medication self-titration on systolic blood pressure in hypertensive patients at high risk of cardiovascular disease: the TASMIN-SR randomized clinical trial. JAMA. 2014;312:799–808. doi: 10.1001/jama.2014.10057 25157723 10.1001/jama.2014.10057

[23] Tucker KL Bowen L Crawford C Mallon P Hinton L Lee MM Oke J Taylor KS Heneghan C Bankhead C. The feasibility and acceptability of self-testing for proteinuria during pregnancy: a mixed methods approach. Pregnancy Hypertens. 2018;12:161–168. doi: 10.1016/j.preghy.2017.11.009 29242046 10.1016/j.preghy.2017.11.009

[24] Tucker KL Sheppard JP Stevens R Bosworth HB Bove A Bray EP Earle K George J Godwin M Green BB. Self-monitoring of blood pressure in hypertension: a systematic review and individual patient data meta-analysis. PLoS Med. 2017;14:e1002389. doi: 10.1371/journal.pmed.1002389 28926573 10.1371/journal.pmed.1002389 PMC5604965

[25] Cairns AE Tucker KL Leeson P Mackillop LH Santos M Velardo C Salvi D Mort S Mollison J Tarassenko L; SNAP-HT Investigators. Self-Management of Postnatal Hypertension: the SNAP-HT trial. Hypertension. 2018;72:425–432. doi: 10.1161/HYPERTENSIONAHA.118.10911 29967037 10.1161/HYPERTENSIONAHA.118.10911

[26] Chung Y de Greeff A Shennan A. Validation and compliance of a home monitoring device in pregnancy: microlife WatchBP home. Hypertens Pregnancy. 2009;28:348–359. doi: 10.1080/10641950802601286 19263287 10.1080/10641950802601286

[27] British Heart Foundation (BHF). How healthy is your diet? Questionnaire. Published April 1, 2012. https://www.bhf.org.uk/informationsupport/publications/health-at-work/health-at-work-how-healthy-is-your-diet-questionnaire

[28] National Institute for Clinical Excellence (NICE). New Guidance 133 (NG133): Hypertension in pregnancy: diagnosis and management. Published June 25, 2019. https://www.nice.org.uk/guidance/ng133 31498578

[29] Stergiou GS Alpert B Mieke S Asmar R Atkins N Eckert S Frick G Friedman B Graßl T Ichikawa T. A universal standard for the validation of blood pressure measuring devices: association for the Advancement of Medical Instrumentation/European Society of Hypertension/International Organization for Standardization (AAMI/ESH/ISO) Collaboration Statement. Hypertension. 2018;71:368–374. doi: 10.1161/HYPERTENSIONAHA.117.10237 29386350 10.1161/HYPERTENSIONAHA.117.10237

[30] Haug EB Horn J Markovitz AR Fraser A Vatten LJ Macdonald-Wallis C Tilling K Romundstad PR Rich-Edwards JW Åsvold BO. Life course trajectories of cardiovascular risk factors in women with and without hypertensive disorders in first pregnancy: the HUNT study in Norway. J Am Heart Assoc. 2018;7:e009250. doi: 10.1161/JAHA.118.009250 30371249 10.1161/JAHA.118.009250 PMC6201453

[31] Tangren JS. Can Blood Pressure Self-Monitoring Improve Postpartum Management of Pregnancy-Associated Hypertension? Hypertension. 2018;72:296–297. doi: 10.1161/HYPERTENSIONAHA.118.11044 29967042 10.1161/HYPERTENSIONAHA.118.11044

[32] World Health organization (WHO): Introduction to drug utilization research. Chapter 6: Drug utilization metrics and their applications. 2003. https://apps.who.int/iris/handle/10665/42627

[33] Fletcher BR Hartmann-Boyce J Hinton L McManus RJ. The effect of self-monitoring of blood pressure on medication adherence and lifestyle factors: a systematic review and meta-analysis. Am J Hypertens. 2015;28:1209–1221. doi: 10.1093/ajh/hpv008 25725092 10.1093/ajh/hpv008

[34] Law MR Morris JK Wald NJ. Use of blood pressure lowering drugs in the prevention of cardiovascular disease: meta-analysis of 147 randomised trials in the context of expectations from prospective epidemiological studies. BMJ. 2009;338:b1665. doi: 10.1136/bmj.b1665 19454737 10.1136/bmj.b1665 PMC2684577

[35] Banegas JR Ruilope LM Williams B. Ambulatory blood pressure and mortality. N Engl J Med. 2018;379:1287–1288. doi: 10.1056/NEJMc1809851 30257163 10.1056/NEJMc1809851

[36] Banegas JR Ruilope LM de la Sierra A Vinyoles E Gorostidi M de la Cruz JJ Ruiz-Hurtado G Segura J Rodríguez-Artalejo F Williams B. Relationship between clinic and ambulatory blood-pressure measurements and mortality. N Engl J Med. 2018;378:1509–1520. doi: 10.1056/NEJMoa1712231 29669232 10.1056/NEJMoa1712231

[37] Pickering TG Shimbo D Haas D. Ambulatory blood-pressure monitoring. N Engl J Med. 2006;354:2368–2374. doi: 10.1056/NEJMra060433 16738273 10.1056/NEJMra060433

[38] Melchiorre K Sutherland GR Baltabaeva A Liberati M Thilaganathan B. Maternal cardiac dysfunction and remodeling in women with preeclampsia at term. Hypertension. 2011;57:85–93. doi: 10.1161/HYPERTENSIONAHA.110.162321 21098311 10.1161/HYPERTENSIONAHA.110.162321

[39] Melchiorre K Sutherland GR Liberati M Thilaganathan B. Preeclampsia is associated with persistent postpartum cardiovascular impairment. Hypertension. 2011;58:709–715. doi: 10.1161/HYPERTENSIONAHA.111.176537 21844489 10.1161/HYPERTENSIONAHA.111.176537

[40] Khalil RA Granger JP. Vascular mechanisms of increased arterial pressure in preeclampsia: lessons from animal models. Am J Physiol Regul Integr Comp Physiol. 2002;283:R29–R45. doi: 10.1152/ajpregu.00762.2001 12069928 10.1152/ajpregu.00762.2001

[41] Noori M Donald AE Angelakopoulou A Hingorani AD Williams DJ. Prospective study of placental angiogenic factors and maternal vascular function before and after preeclampsia and gestational hypertension. Circulation. 2010;122:478–487. doi: 10.1161/CIRCULATIONAHA.109.895458 20644016 10.1161/CIRCULATIONAHA.109.895458

[42] Ghossein-Doha C Peeters L van Heijster S van Kuijk S Spaan J Delhaas T Spaanderman M. Hypertension after preeclampsia is preceded by changes in cardiac structure and function. Hypertension. 2013;62:382–390. doi: 10.1161/HYPERTENSIONAHA.113.01319 23734003 10.1161/HYPERTENSIONAHA.113.01319

[43] Ormesher L Higson S Luckie M Roberts SA Glossop H Trafford A Cottrell E Johnstone ED Myers JE. Postnatal enalapril to improve cardiovascular function following preterm preeclampsia (pick-up):: a randomized double-blind placebo-controlled feasibility trial. Hypertension. 2020;76:1828–1837. doi: 10.1161/HYPERTENSIONAHA.120.15875 33012200 10.1161/HYPERTENSIONAHA.120.15875 PMC7610547

[44] Newell SA Girgis A Sanson-Fisher RW Savolainen NJ. The accuracy of self-reported health behaviors and risk factors relating to cancer and cardiovascular disease in the general population: a critical review. Am J Prev Med. 1999;17:211–229. doi: 10.1016/s0749-3797(99)00069-0 10987638 10.1016/s0749-3797(99)00069-0

[45] Penaloza-Ramos MC Jowett S Mant J Schwartz C Bray EP Sayeed Haque M Richard Hobbs FD Little P Bryan S Williams B. Cost-effectiveness of self-management of blood pressure in hypertensive patients over 70 years with suboptimal control and established cardiovascular disease or additional cardiovascular risk diseases (TASMIN-SR). Eur J Prev Cardiol. 2016;23:902–912. doi: 10.1177/2047487315618784 26603745 10.1177/2047487315618784

[46] Gynaecologists RCoO. Coronavirus (COVID-19) infectoin in pregnancy. RCOG. 2021;1:67

[47] Kitt J Fox R Tucker KL McManus RJ. New approaches in hypertension management: a review of current and developing technologies and their potential impact on hypertension care. Curr Hypertens Rep. 2019;21:44. doi: 10.1007/s11906-019-0949-4 31025117 10.1007/s11906-019-0949-4 PMC6483962

