# Supplementary figures and images for: Short-Term Postpartum Blood Pressure Self-Management and Long-Term Blood Pressure Control: A Randomized Controlled Trial
Source: Hypertension. 2021 Jun 28;78(2):469–79. doi: 10.1161/HYPERTENSIONAHA.120.17101 (PMC8260340; doi:10.1161/HYPERTENSIONAHA.120.17101)

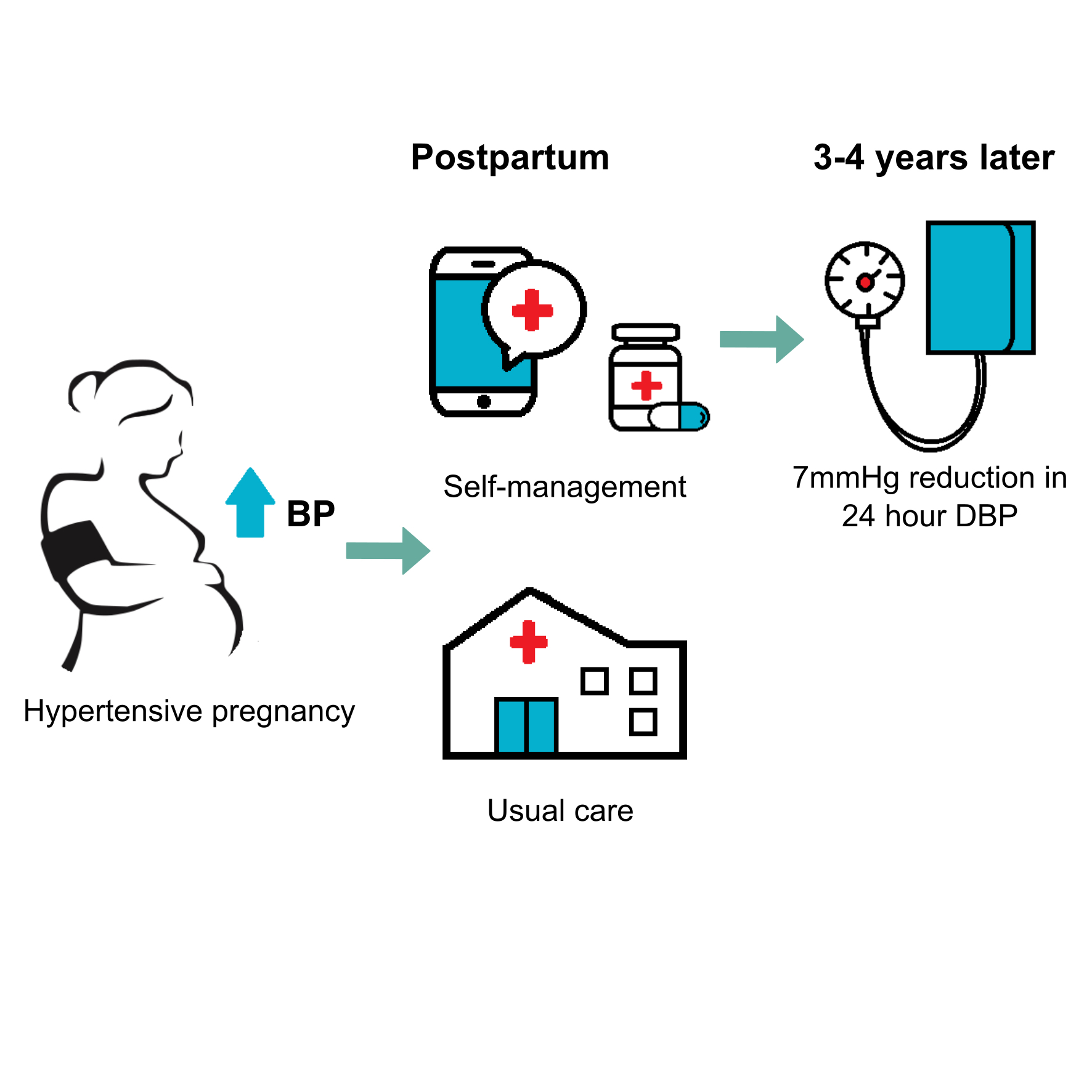

Supplement: Supplementary file 1 [file hyp-78-469-s001.jpg]
