# Supplementary material for: Short-Term Postpartum Blood Pressure Self-Management and Long-Term Blood Pressure Control: A Randomized Controlled Trial
Source: Hypertension. 2021 Jun 28;78(2):469–79. doi: 10.1161/HYPERTENSIONAHA.120.17101 (PMC8260340; doi:10.1161/HYPERTENSIONAHA.120.17101)
Supplement: Supplementary file 2 [file hyp-78-469-s002.doc]

**DATA SUPPLEMENT**

**Title:** Short term postpartum blood pressure management and long term blood pressure control: a randomized controlled trial

**Authors:** Jamie Kitt1 BM BCh MA, Rachael Fox2 BBMed MD, Alexandra Cairns BM BCh MA DPhil3, 4, Jill Mollison PhD3, Holger Burchert MSc1, Yvonne Kenworthy BSc1, Annabelle McCourt MSc1, Katie Suriano1 BSc PhD, Adam J. Lewandowski BSc (Hons) DPhil1, Lucy Mackillop4 BM BCh MA, Katherine Tucker3 BSc (Hons) PhD, Richard McManus*3† MA PhD MBBSand Paul Leeson MB PhD­­*1

*Joint Senior Authors

**Affiliations:**

1Cardiovascular Clinical Research Facility, RDM Division of Cardiovascular Medicine, University of Oxford, Oxford, UK

2University of Melbourne, Melbourne, Australia and Western Health, Melbourne, Australia

3Nuffield Department of Primary Care, University of Oxford, Oxford, UK

4 Nuffield Department of Women's and Reproductive Health, University of Oxford, Oxford, UK

**Corresponding Author:**

†Prof Richard McManus

Nuffield Department of Primary Care, University of Oxford, Radcliffe Primary Care, Radcliffe Observatory Quarter, Woodstock Road, OX2 6GG, UK;

[Richard.McManus@phc.ox.ac.uk](mailto:Richard.McManus@phc.ox.ac.uk);

Tel: +44 (0)1865 617852;

<https://orcid.org/0000-0003-3638-028X>

**Supplemental Tables**

| **Supplementary table S1. Comparison of baseline characteristics at time of enrolment to SNAP-HT for those re-recruited to SNAP-HT Extension vs. those not re-recruited or excluded from SNAP-HT Extension** | | |
| --- | --- | --- |
| **Parameter, unit** | **Re-recruited to SNAP-HT Extension (n=61)*** | **Not re-recruited or excluded from SNAP-HT Extension (n=33)**† |
| Median gestational age at diagnosis, week (IQR) | 35.0 (32.0, 37.6) | 36.0 (32.1, 36.7) |
| Mean age in original study, years±SD | 31.0±5.4 | 32.3±5.0 |
| Parity at original study, n (%) | | |
| 0 | 39 (63.9) | 26 (78.8) |
| ≥ 1 | 22 (36.1) | 7 (21.2) |
| Diagnosis, n (%) | | |
| Preeclampsia | 27 (44.3) | 16 (48.5) |
| Gestational hypertension | 34 (55.7) | 17 (51.5) |
| IMD quintile, n (%) | | |
| First | 26 (42.6) | 15 (45.5) |
| Second | 7 (11.5) | 9 (27.2) |
| Third | 18 (29.5) | 5 (15.2) |
| Fourth | 9 (14.8) | 2 (6.1) |
| Fifth | 1 (1.6) | 2 (6.1) |
| Median duration of antenatal AHT treatment, days (IQR) | 10.0 (6, 20)‡ | 8.0 (4, 20) |
| Mean SBP/DBP at antenatal booking visit, mmHg ± SD | 115.8±12.9/71.5±9.3§ | 122.2±14.4/75.2±9.3 |
| Mean SBP/DBP (readings 2nd and 3rd)|| at baseline in SNAP-HT#, mmHg ± SD | 132.3±14.4/85.5±10.5‡ | 134.1±14.4/87.6±8.7 |
| Mean SBP/DBP (readings 2nd to 6th)** at baseline in SNAP-HT, mmHg ± SD | 132.1±12.6/85.5±9.6‡ | 134.7±12.8/88.3±8.6†† |

AHT indicates antihypertensive; BMI, body mass index; DBP, diastolic blood pressure; IMD, index of multiple deprivation; IQR, interquartile range; SBP, systolic blood pressure and SD, standard deviation.
*61 women include 58 women that were randomized in SNAP-HT, plus 3 women who withdrew from SNAP-HT as per CONSORT.
†33 women not re-recruited include the remaining women that were randomized in SNAP-HT but did not consent to SNAP-HT Extension.
‡n=58, baseline data missing for three individuals from original SNAP-HT data set.
§n=59, two antenatal blood pressure values missing from original SNAP-HT data set.
||Six readings taken at one-minute intervals: 1st reading discarded, mean of 2nd and 3rd readings used. #baseline visit day 1-6 after birth;
**Six readings taken at one-minute intervals: mean of second to sixth readings used;
††n=32, missing sixth blood pressure reading for one individual

| **Supplementary table S2. Univariable linear regression of risk factors for hypertension to determine association with three-year average 24-hour DBP** | | |
| --- | --- | --- |
| **Risk Factor** | **B coefficient (95% CI)** | **P value** |
| Mean age at re-recruitment, years | 0.26 (-0.09, 0.61) | P = 0.14 |
| Parity at re-recruitment | 1.5 (-0.62, 3.60) | P = 0.16 |
| Median BMI at re-recruitment, kg/m2 | 0.05 (-0.20, 0.30) | P =0.68 |
| Activity, hours/day* | |  |
| Sleep | -0.02 (-1.47, 1.42) | P = 0.98 |
| Sedentary | 0.09 (-1.12, 1.29) | P = 0.89 |
| Light | -0.10 (-2.32, 2.11) | P =0.93 |
| Walk | 0.25 (-1.42, 1.92) | P = 0.77 |
| Moderate | -0.58 (-2.79, 1.63) | P = 0.60 |
| Moderate-Vigorous | 0.12 (-2.15, 2.40) | P = 0.91 |
| Vigorous | -3.87 (-30.28, 22.55) | P = 0.77 |
| Current smokers, n | 5.57 (-1.83, 12.96) | P = 0.14 |
| Alcohol use, units/week† | 0.49 (-1.81, 2.78) | P = 0.67 |
| Salt intake, n‡ | 4.59 (1.81, 7.37) | P = 0.002 |
| Mean arm circumference, cm | -0.21 (-0.59, 0.17) | P = 0.28 |
| Mean waist to hip ratio | 11.24 (-12.14, 34.62) | P = 0.34 |

BMI indicates body mass index and DBP, diastolic blood pressure *As measured with Axivity AX3® accelerometers
†Categorised as per Table 3 into 0, 1-7, 8-14 and >15 standard drinks per week
‡Categorised as low, medium and high in accordance with their answers to the British Heart Foundation dietary questionnaire. Low intake was classed as 0-1 positive responses to the salt intake questions, 2-3 as moderate intake and 4 or more answers to the salt questions as high.

| **Supplementary table S3. Summary of results with adjustments for baseline BP (mean of 2ndto 6th readings) and antenatal BP** | | | | |
| --- | --- | --- | --- | --- |
| **Parameter, unit** | **ΔI-C, mmHg (95% CI)*****adjusted for baseline BP (mean 2nd to 6th)**† | **ΔI-C, mmHg (95% CI)*** **adjusted for baseline BP (mean 2nd to 6th)**† **and salt intake at 4 year follow up** | **ΔI-C, mmHg (95% CI)**‡ **adjusted for antenatal BP**§ | **ΔI-C, mmHg (95% CI)**‡ **adjusted for antenatal BP**§ **and salt intakeat 4 year follow up** |
| PRIMARY OUTCOME  Mean 24 hr average DBP||, mmHg ± SD | -7.3 (-10.5, -4.0)#  P < 0.001 | -6.7 (-9.8, -3.6)#  P < 0.001 | -6.9 (-10.3, -3.6)#  P < 0.001 | -6.4 (-9.5, -3.2)#  P < 0.001 |
| SECONDARY OUTCOMES | | | | |
| Mean 24 hr average SBP||, mmHg ± SD | -4.6 (-10.0, 0.9)  P = 0.098 | -4.0 (-9.4, 1.5)  P = 0.15 | -6.1 (-11.1, -1.2)#  P = 0.016 | -5.6 (-10.4, -0.7)#  P = 0.024 |
| Mean diurnal DBP||, mmHg ± SD | -5.1 (-8.4, -1.8)#  P = 0.003 | -4.5 (-7.6, -1.4)#  P = 0.006 | -4.8 (-8.3, -1.4)#  P = 0.007 | -4.2 (-7.5, -1.0)#  P = 0.011 |
| Mean diurnal SBP||, mmHg ± SD | -2.9 (-8.4, 2.5)  P = 0.29 | -2.1 (-7.4, 3.2)  P = 0.43 | -4.1 (-9.3, 1.1)  P = 0.12 | -3.4 (-8.3, 1.6)  P = 0.18 |
| Mean nocturnal DBP||, mmHg ± SD | -7.7 (-11.8, -3.6)#  P < 0.001 | -7.3 (-11.3, -3.3)#  P = 0.001 | -7.5 (-11.6, -3.3)#  P = 0.001 | -7.1 (-11.2, -2.9)#  P = 0.001 |
| Mean nocturnal SBP||, mmHg ± SD | -7.4 (-14.2, -0.6)#  P = 0.034 | -6.9 (-13.8, -0.08)#  P = 0.048 | -8.6 (-15.2, -2.0)#  P = 0.012 | -8.1 (-14.7, -1.5)#  P = 0.017 |
| Mean Clinic DBP (readings 2nd and 3rd), mmHg ± SD | -3.4 (-8.1, 1.2)  P = 0.14 | -3.0 (-7.6, 1.6)  P = 0.19 | -3.5 (-8.1, 1.0)  P = 0.13 | -3.1 (-7.7, 1.4)  P = 0.17 |
| Mean Clinic SBP (readings 2nd and 3rd), mmHg ± SD | -0.1 (-6.4, 6.3)  P = 0.98 | 0.5 (-5.8, 6.9)  P = 0.87 | -1.3 (-7.4, 4.8)  P = 0.67 | -0.7 (-6.8, 5.3)  P = 0.81 |
| Mean Clinic DBP (readings 2nd to 6th), mmHg ± SD | -2.8 (-6.7, 1.2)  P = 0.16 | -2.5 (-6.5, 1.4)  P = 0.21 | -2.9 (-6.8, 1.0)  P = 0.14 | -2.6 (-6.6, 1.3)  P = 0.18 |
| Mean Clinic SBP (readings 2nd to 6th), mmHg ± SD | -0.04 (-5.6, 5.6)  p = 0.99 | 0.3 (-5.3, 6.0)  p = 0.91 | -1.0 (-6.5, 4.4)  P = 0.70 | -0.7 (-6.1, 4.8)  P = 0.81 |

ΔI-C indicates difference between intervention and control groups; DBP, diastolic blood pressure; SBP, systolic blood pressure and SD, standard deviation.
*n=58, three missing baseline blood pressure measurements from control group
†mean difference adjusted for baseline blood pressure values in SNAP-HT, day 1-6 after birth.
‡n=59, two missing antenatal blood pressure measurements from control group
§mean difference adjusted for antenatal booking visit blood pressure values in SNAP-HT
||measured by SPACELAB 90217 ambulatory blood pressure monitor
#95% CI around adjusted difference does not cross zero

| **Supplementary table S4. Sensitivity analysis with the three non-randomised participants excluded** | | |
| --- | --- | --- |
| **Parameter, unit** | **ΔI-C, mmHg (95% CI) adjusted for baseline BP (mean 2nd and 3rd)*** | **ΔI-C, mmHg (95% CI) adjusted for baseline BP (mean 2nd and 3rd)* and salt intake at 4 year follow up** |
| PRIMARY OUTCOME Mean 24 hr average DBP†, mmHg ± SD | -8.1 (-11.2, -4.9) ‡ P < 0.001 | -7.4 (-10.5, -4.4) ‡ P < 0.001 |
| SECONDARY OUTCOMES | | |
| Mean 24 hr average SBP†, mmHg ± SD | -5.2 (-10.6, 0.3) P = 0.062 | -4.6 (-10.0, 0.9) P = 0.01 |
| Mean diurnal DB†, mmHg ± SD | -6.0 (-9.2, -2.8) ‡ P < 0.001 | -5.3 (-8.4, -2.2) ‡ P = 0.001 |
| Mean diurnal SBP†, mmHg ± SD | -3.3 (-8.7, 2.2) P = 0.23 | -2.5 (-7.9, 2.9) P = 0.36 |
| Mean nocturnal DBP†, mmHg ± SD | -8.7 (-12.7, -4.6) ‡ P < 0.001 | -8.3 (-12.4, -4.2) ‡ P < 0.001 |
| Mean nocturnal SBP†, mmHg ± SD | -8.7 (-15.4, -2.0) ‡ P = 0.012 | -8.4 (-15.2, -1.6) ‡ P = 0.017 |
| Mean Clinic DBP (readings 2nd and 3rd), mmHg ± SD | -3.7 (-8.4, 1.1) P = 0.13 | -3.1 (-7.9, 1.6) P = 0.20 |
| Mean Clinic SBP (readings 2nd and 3rd), mmHg ± SD | -0.30 (-6.7, 6.1) P = 0.93 | 0.32 (-6.1, 6.8) P = 0.10 |
| Mean Clinic DBP (readings 2nd to 6th), mmHg ± SD | -2.9 (-7.0, 1.2) P = 0.16 | -2.6 (-6.8, 1.5) P = 0.21 |
| Mean Clinic SBP (readings 2nd to 6th), mmHg ± SD | -0.03 (-5.6, 5.6) P =1.0 | 0.32 (-5.4, 6.0) P =0.91 |

ΔI-C indicates difference between intervention and control groups; DBP, diastolic blood pressure; SBP, systolic blood pressure and SD, standard deviation.
*mean difference adjusted for baseline blood pressure values in SNAP-HT, day 1-6 after birth.
†measured by SPACELAB 90217 ambulatory blood pressure monitor
‡95% CI around adjusted difference does not cross zero
